# Adolescence between biology and culture a perspective on the crisis of symbolization

**DOI:** 10.3389/fpsyg.2022.932863

**Published:** 2022-09-08

**Authors:** Stefano Carta, Stefania Cataudella

**Affiliations:** Department of Pedagogy, Psychology, Philosophy, University of Cagliari, Cagliari, Italy

**Keywords:** adolescence, identity, psychopathological disorders, crisis, Oedipus complex, narcissism, anthropology, ethnology

## Abstract

One way to conceptualize human life is to describe it as a process through which the biological body is progressively transformed into a psychological one through its mentalization and symbolization. This process occurs through the relational field, which begins with caregiver-infant proto-conversations and develops through adolescence into the ongoing complex interpersonal relational network we call society and culture. The essence and the problems of adolescents are intricately tied to the social and cultural contexts in which they experience life. Therefore, adolescence cannot be understood if all the levels that it expresses (biological, psychological cultural/social) are not taken into consideration. We identify three psycho-historical phases through which adolescence has changed in the past century: (1) Oedipal; (2) Narcissistic; and (3) Post-narcissistic. In this last phase due to the psychological and historical failure of the narcissistic ideals, the ideal is mingling with the real in a wholly new way. This process has overturned Erikson's paradigm: identity, opposed and defined by a dichotomic otherness, must be transformed into a fluid integration of similarities and differences negotiated and developed through empirical interpersonal intersubjective experiences. This, in our perspective, is a possible key to understand the rapid change in the nature of consciousness, selfhood, and gendering in today's western world, together with some important psychopathological disorders which describe the new creative challenges of today's adolescents.

## Introduction

It is possible to say that human life is a process through which the biological body is progressively transformed into a psychological one through its mentalization and symbolization (Bion, 1962). This process occurs through the relational field, which begins with caregiver–infant proto-conversations and develops through adolescence into the ongoing, complex, and interpersonal relational network we call society and culture. In this sense, Anthropology is the *complementary* side of a whole in which the other aspect is Psychology (Devereux, 1978). Within this perspective, the essence and the problems of adolescents are tied to the social and cultural contexts in which they experience life (Erikson, 1959; Coleman, 1974; Lerner and Foch, 1987; Dahl et al., 2018; Hurrelmann and Quenzel, 2018; Israel et al., 2021). Therefore, adolescence cannot be understood if all the levels that it expresses (biological and psychological cultural/social) are not taken into consideration (Sawyer et al., 2018; Worthman and Trang, 2018).

Adolescence's developmental tasks (Lancini, 2019; Lancini et al., 2020), as highly complex as they are, define adolescence as a real-time of transformation, so pervasive as to be described as a second birth (Blos, 1962, 1967). Erikson (1968) somehow considered adolescence the barycenter of the subject's development, from the beginning of postnatal life (the Freudian “primary narcissism” and the “oral stage”) to old age and the completion of the life cycle by finding meaning in its very finitude. Erikson also theorized that adolescence was essentially a critical process–i.e., somehow a de-integration of the previous infantile state of the self–to obtain a more mature self, now integrated into a developed self-reflective awareness, and subject/object constancy (Mahler et al., 1975)–a self-provided with what Erikson called “identity.” The critical nature of this process and its challenges, for many aspects such as the deeply ambiguous and disconcerting nature of the middle phase of the anthropological rites of passages as described by Van Gennep (1961), are described by Erikson as a “crisis” which may be resolved at the positive end of the process, or which may end in a fragmented psycho-social organization (confusion and diffusion of identity).

We would like to re-formulate Erikson's view. In fact, we think that today both concepts of crisis and identity have become critical themselves. From the consideration that the child at the end of adolescence will become an active agent of his/her cultural (symbolic), social, economic, and historical world, we maintain that adolescence is not only a process that happens through interpersonal time (and that, therefore, may be studied through a purely psychological lens) but that it also takes place within a social, economic, anthropological, and *historical* time (and that, therefore, needs an ethno-psychological perspective). The point is that the same applies to Erikson's theory itself, as we think that today—at least in the capitalistic West—the contemporary empirical form of “identity” is profoundly different from Erikson's time and that this difference implies also a revision of the concept of “crisis,” as Erikson conceptualized it. In a few words, identity and crisis themselves might be a historically determined form of the many ways the structural and representational self is organized.

In our opinion, from the middle of the 1960s to the present time adolescence and, therefore, “identity” and “crisis” have undergone a very deep transformation. A point of reference for this is Deleuze and Guattari's *Anti-Oedipus* (Deleuze and Guattari, 1977), in which the authors analyzed the reformulation of the organization of the personality in the West as wholly embedded within history and, therefore, capitalism. In fact, capitalism is the form of the most pervasive contemporary *mythology* that organizes the totality of human life in post-modernity (for an interpretation of mythology see, for instance, Jung, 1951), and which encompasses narratives that symbolize and organize all levels of life from its embodied forms—such as sex, genders, and procreation—all the way to the social, religious, and cultural levels. Under this perspective, Deleuze and Guattari's position seems very pertinent, indeed.

Within a psycho-historical perspective we individuate three main phases: (1) Oedipal; (2) Narcissistic; and (3) Post-Narcissistic.

## The Oedipal phase

Before the 1960s, the main organizing mental–and therefore social and anthropological (hence mythical) structure, at least for the western's mind, was the Oedipus complex. Just to sketch our argument: in the Oedipus complex, history is (a) conservative, as the child must identify with the Father and therefore carry his value-system, under the form of Super-Ego, toward the next generation; and (b) repressive, as the polymorphic and “perverse” (Freud, 1905) nature of the unconscious (the infinitely creative and alive *body without organs*, as defined by Deleuze and Guattari (1977) is confined within rigid norms under the threat of castration—i.e., the absolute loss of psychic life. Erikson was writing precisely during this time when *historical* identity was under huge attack (a crisis) by the “Beat generation.” In fact, during the revolution of the 60s, we notice a migration of the ideal from the Father to the Child. Before the 1960s, the empirical representation of the self could provide *stability and recognition* to the personality by granting not only its *regulative* function but because of its transgenerational stability derived from the identification with the Father, also the illusion of its *constitutive* reality (Kant, 1967), which psychoanalysis (Freud, 1905) and analytical psychology (Jung, 1951) had already definitively dismantled by proving the intrinsic dissociability of the psyche. For Erikson (1950, 1968), the synthetic and regulative function of the ego would grant stability, and recognition together with a highly stable—apparently constitutive—*identity*, which means: the possibility to constitutively belong to a specific psycho-social category, *stable* in biographical, social and historical time—a category whose borders, once established—had to remain very well defined and fixed. Such an identity would automatically define the other as a wholly Other. Erikson's world was a world based on static and well-defined identities express by nouns, and not on fluid relationships expressed by adjectives and verbs, a world destined to change under a complex set of historical conditions, among which: the two world wars, the ever-increasing pace of capitalism, materialism, individualism, and the subordination of every possible difference to that of the relationship between the subject as a consumer and his objects as commodities, and the logarithmic acceleration of time.

## The narcissistic phase

The revolution of the 60s demolished the super-egoic authority of the Father and therefore, broke transgenerational continuity, while historical changes dramatically accelerated (Lévi-Strauss, 1967). This meant the *migration of the ideal from the Father to the Child*, and the subsequent birth of psycho-social mythology whose essential feature was narcissism (Sobo, 1977). For this myth, the Child, and with-it youth, consumption, creativity, change, manic-like lifestyles, and transgression become the basic organizers of identity. A narcissistic identity for which the adolescent has the illusion that he/she must be a uniquely precious being (who he/she is, indeed. The question is what the adolescent will do with the *opposite* values: his/her “normality”). In this phase, the Western adolescent (and hence the future adult) believes that he/she may fabricate his/her own values; that he/she is *already* mature before any painstaking process of learning. The fact that learning is necessary to pursue an ideal that, differently from what the subject believes, is *not yet real, and will never be*, is negated. In Freudian terms, this is due to the unresolved Oedipal complex and the impossibility to enter into latency. The Child has the illusion to be already an adult. The ideal seems real.

In this narcissistic historical phase, sexuality is liberated; the myths of “authenticity” and creativity are claimed, although the tragic illusion (which will be progressively clear in the next phase) of the *right* to be adored and admired (by the Oedipal mother) remains an illusion; an illusion which the fundamental capitalistic myth will use for its own structural purposes: consumption and commodification. In this scene, while the adolescent tries to realize his/her “identity,” more than getting to love an exogamic—a real—object, he/she wishes to be loved by the object. Ethics—which was a central feature of the previous Oedipal/Superegoic phase—is now substituted by a narcissistic exhibitionism and, therefore, with esthetics. This is the time in which bulimia comes into the world scene for the first time ever, probably as an ethno-psychological tragic cry that something, in the liberation of women from the phallic domination is failing and should be re-thought in much more radical terms (Anderson-Fye, 2018).

This narcissistic historical phase was bound to fail, together with its cocky and yet extremely fragile, insecure “identity,” which had lost its symbolic reference to the Father. In fact, this phase-specific form of identity formation cannot mark the completion of adolescence, as it was with Erikson, but will actually infinitely *prolong* it by transforming adolescence itself in a permanent, positive pseudo-value for every age. Instead of a child becoming an adult through adolescence, we witness an eternally pseudo-adolescent ideal adult. Within this sense of identity, everyone wishes to remain young and “creative”; everyone will claim an enslaving and impossible hyper-sexuality (which, in the capitalistic myth will be soon pornographically commodified and used as a form of consumption), an eternal youth, together with the “right” to affirm the subject's opinions even if they have no real ground, logic, rationality, or sophistication. In this context, social media are the places in which everyone may *tell without ever listening* (McCain and Campbell, 2018). Even if there is no real knowledge, expertise, or ground to justify these assertions. Once again: talent is narcissistically given for granted, even if it does not exist, and the process of learning (i.e., living, growing up) is ignored. Within this psycho-anthropological milieu, how can an adolescent realize the tasks of such a complex process? Within this picture, how can we define “crisis” as referred to as adolescence if the whole ideal regarding how life should be has become adolescent-like? The crisis of what once was adolescence becomes the existential crisis of every age for everyone.

## The post-narcissistic phase

The third phase is the present one. As with all developmental cycles, the end of the previous narcissistic phase is the result of its own acme, for which, paradoxically, it is the adults who have absorbed many values of adolescence. In this historical phase, a large multiplicity of factors, which involve the structure of the western self, disrupt the very core of what we so far called “identity.” Among these factors we may remember: the hyper fragmentation of social life and the transgenerational discontinuity, which entails the idealization of present and future and the de-idealization of the past (which carries a sense of being uprooted from the “world of the ancestors” and makes it much harder to recognize the pervasive transgenerational nature of psychological conflicts); globalization—i.e., the infinite flow of equivalent commodities, among which we include humans, neutralized by their being measured exclusively by their economic value as producers/consumers; the possibility to live *apparently* infinite lives in what we now know as the “virtual world” (unknown in history before today), while these lives risk to remain virtual anyway and, therefore, are an obstacle to the fundamental goal of adolescence of *realizing* (knowing / making real) its ideals; the compulsive, desperate way to try to exist as socialized individuals (i.e., to stably exist through compulsive requests of mirroring the adolescent's idealized narcissism by others) as we may notice from the explosion of the phenomenon of the *selfie*; the progressive empirical realization that the idealized Child actually lives a *less* creative and meaningful life, bound to commodification and consumption (hence, that the subject of experience is actually subjected *to* his/her commodified objects); the looming realization that he/she will *not* be admired as he/she needs, since everyone—as it always happens in adolescence—is looking for the same recognition by the others (Chopik and Grimm, 2019).

To this progressive realization of the betrayed promises that the idealized narcissistic Child pursued is the realization of the climate crises and, generally, of the catastrophic results of the last decades of the Anthropocene. The world is not the oyster in which the narcissistic Child will shine. An anomic trait of this historical phase is the hiatus between an illusion of being an ideal Child and the absurd cutthroat competitiveness which children, adolescents, and post-adolescents must face throughout their schooling, and where cooperation (which, again, promotes social fragmentation, and intrinsic solitude) seems not to be convenient. Here we see the covert, hugely destructive revenge of the Father toward his children.

In fact, the previous narcissistic investment of the idealized Child had created the conditions for a structural shrinking of the former psychosocial categories of identity, which were able to clearly and stably define “how to be what.” This phenomenon, for which identity categories become less and less inclusive, is very coherent with the progressive molecularization and fragmentation of the social world (which carries with itself a fragmentation of the nuclear family itself). Now, the adolescent becomes always more and more a self-defined, nuclear subject, while the identity categories, without the organizing role of the Father, become extremely fluid.

In this phase–the present post-narcissistic one—the “identity” of this atomized subject, based on his/her narcissistic idealization (I am special) *cannot be considered an identity in the old sense anymore*. In fact, in this psycho-historical phase, we witness the end of identity as Erikson had envisioned. The social and psychological fluidity (Bauman, 2000, 2009) coupled with the extreme narcissistic individualism (a sort of ego-centrism) cannot any longer resort to already given categories (destroyed together with the Oedipal Father) into which identify. Now, identity refers not to belonging to an already given social, personal, or sexual top–down category (as it happened in the Oedipal historical phase), but to *an empirical bottom–up cluster*. If the Oedipal category of the pre-narcissistic phase was a psycho-social formation derived from the idealized Father, now the adolescent's “identity” resembles the aggregation of specific, empirical features that the individual—the subject—collects bottom–up through his/her own intersubjective, interpersonal, and social life. Through this path, he/she will aggregate into a cluster of his/her characteristics *and only at this point* look around to see who could be defined in the same way, and who could belong to the same cluster. This process is producing enormous stress on language, its nouns, and pronouns (She, He, It, … How many pronouns will be needed? What will the balance between the narcissistic atomized identity and the fluidity of belonging to clusters be?).

We are witnessing a powerful process of hybridization and creolization, in which differences and similarities mingle and form *fluid identities*, which, paradoxically deconstruct what is identical and do not denote anything as identical anymore (Remotti, 2019). As highlighted by Lemma (2015, 2018) freedom of choice and the right to self-realization emerge as guiding principles. Indeed, it could be argued that nowadays we are expected to present ourselves as biographically flexible and open to change. This freedom of choice finds its synthesis in the ability to customize one's body—a trend modeled on consumer choices under the dominance of the neo-liberal consumerist concept with the attendant risk that identity is based on what the author herself defines as “acquisitive imitations” where imitation trumps identification. It seems that today, what Erikson called “crisis” has actually become the immanent device of this fluidity. What was critical then, is normal and constitutive now. Obviously, the challenges of this peculiar nature of adolescence are, as usual, very great. Yet, they seem to be very different from those of the Erikson's Oedipal phase, and even those of the previous narcissistic phase. The possibility to find and in dwell within these multiple, changing clusters may not only produce a less difference, but also less polarized and conflictual personality structure and society. It may also produce identity disorders and a feeling of non-continuity of one's personal biography (something that we have witnessed in the progressive transformation of psycho-pathologies in the last 60 years), or dangerous potential phenomena of superficial forms of identification in search for a more stable identity definition.

One thing is evident among all: what for Freud was the fundamental, essential, oppositional difference—the difference that granted identity and, therefore, also conflict, scapegoating, and splitting—the *difference between the sexe*s—is now about to explode under the phenomenal deconstruction of identity and crisis and the multiplication of psycho-socio *genders*. Especially, this phenomenon of gender fluidity seems to represent the multiplication of identity clusters and the wholly new way to build bottom-up, a (fluid) identity through biographical time.

## Clinical conclusions

Through our perspective, we tried to analyze the historical transformation of adolescence—itself a psychological developmental process embedded in socio-cultural history. The pivotal psychological constructs that we have identified are as follows: (a) the transformation of the ideal and its “migration” from the Father to the Child; (b) the deconstruction of what we usually call “identity,” together with; and (c) the conceptualization of the adolescent process as a passage that needs to be structured by rituals as those of the anthropological rites of passage.

This view of ours implies some corollary considerations: (1) the fate of the transformations of adolescence, its ideals and identity, as we have described it, may produce defensive movements of retreat from interpersonal involvement, which may produce phenomena such as that of the so-called hikikomori; (2) the increase of internalized symptoms involving the self, and the body (self), where aggressivity may be conveyed, with or without acting outs (such as self-harming), instead of the frequent externalizing symptoms (or just manifestations) of the adolescent's protest against society; (3) the difficulty to balance the idealizing ideals toward the object world (such as falling in love) and the narcissistic idealization of one's self (wanting to be admired), which may produce a difficult harmonization of sexual and attachment motivations and the decrease of genital sexual involvement; and (4) the progressive increase of the once (illusory) unity of identity at all levels, from the social/professional one, to those aspects that involve gender.

Referring to the Jungian point of view, we think that within the adolescent's clinical setting not only it is undesirable to promote a transference related to the adolescent's infantile history, but that this would go against the very essence of his/her psychological development (this is also the position of Pietropolli-Charmet et al., 2010). In fact, never as in adolescence is it clear that (all) psychological processes are teleological and that it is useful to consider memories and past events only if we frame them as causes-for-intrinsic aims. The past, infancy, and childhood must be seen as preparatory conditions for something *future*. This “future”—the temporal place where the self will realize itself in the world—happens, in its purest form, within adolescence. Seen this way, the process of adolescence *needs* not parents, or “experts,” who, already “knowing” do not express any future anymore, but figures such as *mentors*, who are called to initiate the adolescent into the adult world and a coherent psychological organization. This mentor might be called to perform the same functions that through the rites of passage initiate the person into a renewed and more encompassing form of life. Not transference, but the therapeutic alliance is therefore pivotal. Under this respect, the role of the psychotherapist (or also of the non-parental adult) acquires in adolescence a fundamental transitional role, for which knowledge of anthropology and ethnology might be a necessary requisite.

Such a tension toward the future describes the adolescent as the subject engrossed with the quintessential human need: to symbolize effects. It should be obvious that “symbolization” cannot but refer to a psychological activity of the mind which expresses what we call “culture” at any level we may describe it—from the interpersonal way a caregiver interacts with her/his child, to social life within history. Such a perspective, which joins symbolization, relationship, culture, and teleology, involves the adult, but in its purest and most intense form, especially the adolescent. It is adolescence's fundamental nature. This makes it imperative that the psychotherapist acquires an anthropological lens through which he/she may look at the patient, who is wholly engrossed in the very human attempt to symbolize, actualize within the relational world, and dialectically fit within his/her anthropological world his/her emotional (hence also bodily) experience of himself/herself. No reductive psychological theories or clinical approaches, which purely psychologize or biologize the adolescent's challenges, or which reduce the intrinsic creative and open-ended nature of symbolization, can really help the adolescent. Actually, he/she will rightfully resist them.

In the present times more than ever—times in which the Father must be recreated often through what we may call the *inversion of the Shadow*—for which the “positive” traits of kindness, sensibility, love, curiosity, etc., are hidden under “negative” traits, the adolescent will challenge his/her therapist in order to check whether he/she is taking his/her matter as seriously as he/she is: the matters being nothing less than extracting meaning out of life and transcending its tragic, conflictual aspects.

A last clinical issue involved with the present historical situation has to do with “identity,” and therefore, the sense of one's continuity in time and space. Today more than ever, the clinician must not confuse the specific forms within which the adolescent tries to recognize his/her own selfhood with the synthetic activity of the mind. In the past, it was possible to conflate the synthetic activity of the mind with the specific contents and identification that the mind tries to synthesize. In fact, it was possible for someone to be continually, personally, and socially sure “to coherently be a layer,” or even “a man/woman.” Today this is not so, as the plural, possible personal/social contents of one's identity are much more fluid and, often, fragmented. Therefore, the clinician is called to *never conflate the synthesizing ego with its contents, while recognizing, holding, and validating its activity and continuity*. The stabilization of the adolescent's identifications, and therefore of an *implicit, yet impossible to make explicit sense of identity*, will come with time.

## Author contributions

Both authors listed have made a substantial, direct, and intellectual contribution to the work and approved it for publication.

## Conflict of interest

The authors declare that the research was conducted in the absence of any commercial or financial relationships that could be construed as a potential conflict of interest.

## Publisher's note

All claims expressed in this article are solely those of the authors and do not necessarily represent those of their affiliated organizations, or those of the publisher, the editors and the reviewers. Any product that may be evaluated in this article, or claim that may be made by its manufacturer, is not guaranteed or endorsed by the publisher.

## References

[B1] Anderson-Fye E. R. (2018). “Cultural influences on body image and eating disorders,” in The Oxford Handbook of Eating Disorders, eds W. S. Agras, and A. Robinson (Oxford: Oxford University Press), pp. 187–208. 10.1093/oxfordhb/9780190620998.013.9

[B2] Bauman Z. (2000). Liquid Modernity. Cambridge: Polity Press.

[B3] Bauman Z. (2009). Intervista Sull'identità. Bari: Laterza.

[B4] Bion W. W. (1962). Learning from Experience. Lanham: Rowman and Littlefield.

[B5] Blos P. (1962). On Adolescence—A Psychoanalytic Interpretation. Detroit, MI: Free Press.

[B6] Blos P. (1967). The second individuation process of adolescence. Psychoanal. Study Child 22, 162–186. 10.1080/00797308.1967.11822595 5590064

[B7] Chopik W. J. Grimm K. J. (2019). Longitudinal changes and historic differences in narcissism from adolescence to older adulthood. Psychol. Aging 34, 1109–1123. 10.1037/pag0000379 31804115

[B8] Coleman J. C. (1974). Relationships in Adolescence. London: Routledge and Kegan Paul.

[B9] Dahl R. E. Allen N. B. Wilbrecht L. Suleiman A. B. (2018). Importance of investing in adolescence from a developmental science perspective. Nature 554, 441–450. 10.1038/nature25770 29469094

[B10] Deleuze G. Guattari F. (1977). Anti-Oedipus: Capitalism and Schizophrenia. New York, NY: Viking Press.

[B11] Devereux G. (1978). “Two types of modal personality models,” in Ethnopsychoanalisis: Psychoanalisis and Anthropology as Complementary Frames of Reference (Berkeley, CA: University of California Press), 113–135.

[B12] Erikson E. H. (1950). Childhood and Society. New York, NY: WWN.

[B13] Erikson E. H. (1959). Identity and the Life Cycle. Madison, WI: International University Press.

[B14] Erikson E. H. (1968). Identity Youth and Crisis. New York, NY: WW Norton.

[B15] Freud S. (1905). “*Tre sagggi sulla teoria* sessuale,” in Drei Abhandlungen zur Sexualtheorie 1905, ed O. Freud (Torino: Bollati Boringhieri).

[B16] Hurrelmann K. Quenzel G. (2018). Developmental Tasks in Adolescence. London: Routledge 10.4324/9780429452055

[B17] Israel A. C. Malatras J. W. Wicks-Nelson R. (2021). Abnormal Child and Adolescent Psychology. New York, NY: Routledge. 10.4324/9780429286896

[B18] Jung C. G. (1951). Introduction to a Science of Mythology: The Myth of the Divine Child and the Mysteries of Eleusis. London: Routledge and Kegan Paul.

[B19] Kant I. (1967). Critica Della Ragion Pura. Torino: UTET.

[B20] Lancini M. (2019). Il Ritiro Sociale Negli Adolescenti. La Solitudine di una Generazione Iperconnessa. Milano: Raffaello Cortina.

[B21] Lancini M. Cirillo L. Zanella T. (2020). L'adolescente. Psicopatologia e Psicoterapia Evolutiva. Milano: Raffaello Cortina.

[B22] Lemma A. (2015). Psychoanalysis in times of technoculture: some reflections on the fate of the body in virtual space. Int. J. Psychoanal. 96, 569–558. 10.1111/1745-8315.12348 26173880

[B23] Lemma A. (2018). Trans-itory identities: some psychoanalytic reflections on transgender identities. Int. J. Psychoanal. 99, 1089–1106. 10.1080/00207578.2018.1489710 33951791

[B24] Lerner R. M. Foch T. T. (1987). Biological–Psychosocial Interactions in Early Adolescence: An Overview of the Issues. London: Routledge.

[B25] Lévi-Strauss C. (1967). Razza e Storia e Altri Studi di Antropologia. Milano: Einaudi.

[B26] Mahler M. Pine F. Bergman A. (1975). The Psychological Birth of the Human Infant. New York, NY: Basic Books.

[B27] McCain J. L. Campbell W. K. (2018). Narcissism and social media use: a meta-analytic review. Psychol. Pop Media Cult. 7, 308–327. 10.1037/ppm0000137

[B28] Pietropolli-Charmet G. Bignamini S. Comazzi D. (2010). Psicoterapia Evolutiva Dell'adolescente. Milano: Franco Angeli.

[B29] Remotti F. (2019). Somiglianze. Una via Per la Convivenza. Bari: Laterza.

[B30] Sawyer S. M. Azzopardi P. S. Wickremarathne D. Patton G. C. (2018). The age of adolescence. Lancet Child Adolesc. Health 2, 223–228. 10.1016/S2352-4642(18)30022-1 30169257

[B31] Sobo S. (1977). Narcissism as a Function of Culture. Psychoanal. Study Child 32, 155–172. 10.1080/00797308.1977.11822337 918214

[B32] Van Gennep A. (1961). The Rites of Passage. Chicago, IL: University of Chicago Press. 10.7208/chicago/9780226027180.001.0001

[B33] Worthman C. M. Trang K. (2018). Dynamics of body time, social time and life history at adolescence. Nature 544, 451–457. 10.1038/nature25750 29469099

